# Semaglutide as a promising treatment for hypothalamic obesity: a six-month case series on four females with craniopharyngioma

**DOI:** 10.1007/s11102-024-01426-8

**Published:** 2024-08-01

**Authors:** Erlend Gjersdal, Liva Bundgaard Larsen, Kåre Schmidt Ettrup, Peter Vestergaard, Eigil Husted Nielsen, Jesper Scott Karmisholt, Hermann L. Müller, Jakob Dal

**Affiliations:** 1 Department of Endocrinology, Aalborg University hospital, Aalborg, 9000 Denmark; 2 Randers Regional hospital, Medical department, Randers, Denmark; 3 Department of Neurosurgery, Aalborg University hospital, Aalborg, 9000 Denmark; 4 Department of Pediatrics and Pediatric Hematology / Oncology, University Children’s Hospital, Carl von Ossietzky Universität Oldenburg, Klinikum Oldenburg AöR, Oldenburg, 26133 Germany

**Keywords:** Hypothalamic obesity, Craniopharyngioma, GLP1RA, Semaglutide, TFEQ

## Abstract

**Purpose:**

Patients with hypothalamic pathology often develop hypothalamic obesity, causing severe metabolic alterations resulting in increased morbidity and mortality. Treatments for hypothalamic obesity have not proven very effective, although the glucagon-like peptide-1 receptor agonist semaglutide has been shown to have positive effects. We examined semaglutide’s effect on weight loss in a sample of patients with hypothalamic obesity.

**Methods:**

Four female patients with hypothalamic obesity resulting from treatment of craniopharyngiomas were treated with semaglutide for six months. Whole Body Dual-energy x-ray absorptiometry scans were performed, and blood samples drawn at baseline and after six months. Semaglutide dosages were increased monthly along with tracking of body weight and eating behavior (Three Factor Eating Questionnaire, TFEQ-R18).

**Results:**

BMI was reduced in all cases, with an average of 7.9 BMI (range: 6.7 to 10.1) corresponding to a weight loss of 17.0% (range: 11.3–22.4%) or 20.2 kg (range 16.2 kg to 23.4 kg). We found a comparable reduction in total fat mass (17.2%, *p* = 0.006) and lean mass (16.0%, *p* = 0.05), whereas bone mass was unchanged (2.6%, *p* = 0.12). All cases reported an increase in energy levels, improved mobility and physical activity. Unfavorable eating behaviors were reduced after 1 month of treatment (emotional eating − 41 points, *p* = 0.02, uncontrolled eating − 23 points, *p* = 0.11). HbA1c and total cholesterol were significantly reduced (*p* = 0.014 for both).

**Conclusion:**

Semaglutide is a promising and safe treatment option for HO, that improves eating behavior, reduces weight, and improves metabolic markers.

## Introduction

Hypothalamic obesity (HO) is characterized by abnormal weight gain due to structural damage to the hypothalamus, either caused by a pathological lesion, e.g. a craniopharyngioma (CP), or following surgical or radiological intervention [1]. As a consequence, the neural pathways responsible for the regulation of feeding behavior, satiety and energy expenditure, can be disrupted, thus affecting overall energy balance. This disruption often leads to hyperphagia, insulin and leptin resistance, rapid weight gain and fat accumulation, leading to severe obesity in most patients [2, 3]. HO is associated with considerable metabolic and psychological complications as a consequence to the weight gain with increased risks of cardiovascular morbidity, reduced quality of life and increased mortality [4].

Managing HO presents challenges due to the limited effectiveness of lifestyle and pharmacological interventions [5]. Glucagon-like peptide-1 receptor agonists (GLP1-RA), are efficient drugs when used in the treatment of obesity [6–8], and seems to be a promising treatment of HO due to their multifaceted mechanism of action, which extends beyond the affected hypothalamic regions [9]. Results in trials using earlier GLP-1RA such as exenatide have been varying [5, 10–12], with relatively low effect as compared to placebo. However, a recent case report demonstrated a marked body weight reduction and improved metabolic parameters using semaglutide [13]. Semaglutide differs from other GLP-1RA in its longer half-life and a more potent effect on weight loss. It activates different areas of the central nervous system compared to liraglutide and it is suggested to cross the blood-brain barrier more effectively, which may enhance its central anorectic effects. These effects include modulation of appetite and satiety centers in the hypothalamus, leading to a reduction in food intake and improved eating behaviors. This is supported by evidence indicating that semaglutide engages neural circuits involved in energy balance and food reward more robustly than liraglutide [14, 15].

Our case series aimed to document the effects of semaglutide treatment in four patients with HO.

## Materials and methods

**Fig. 1:**
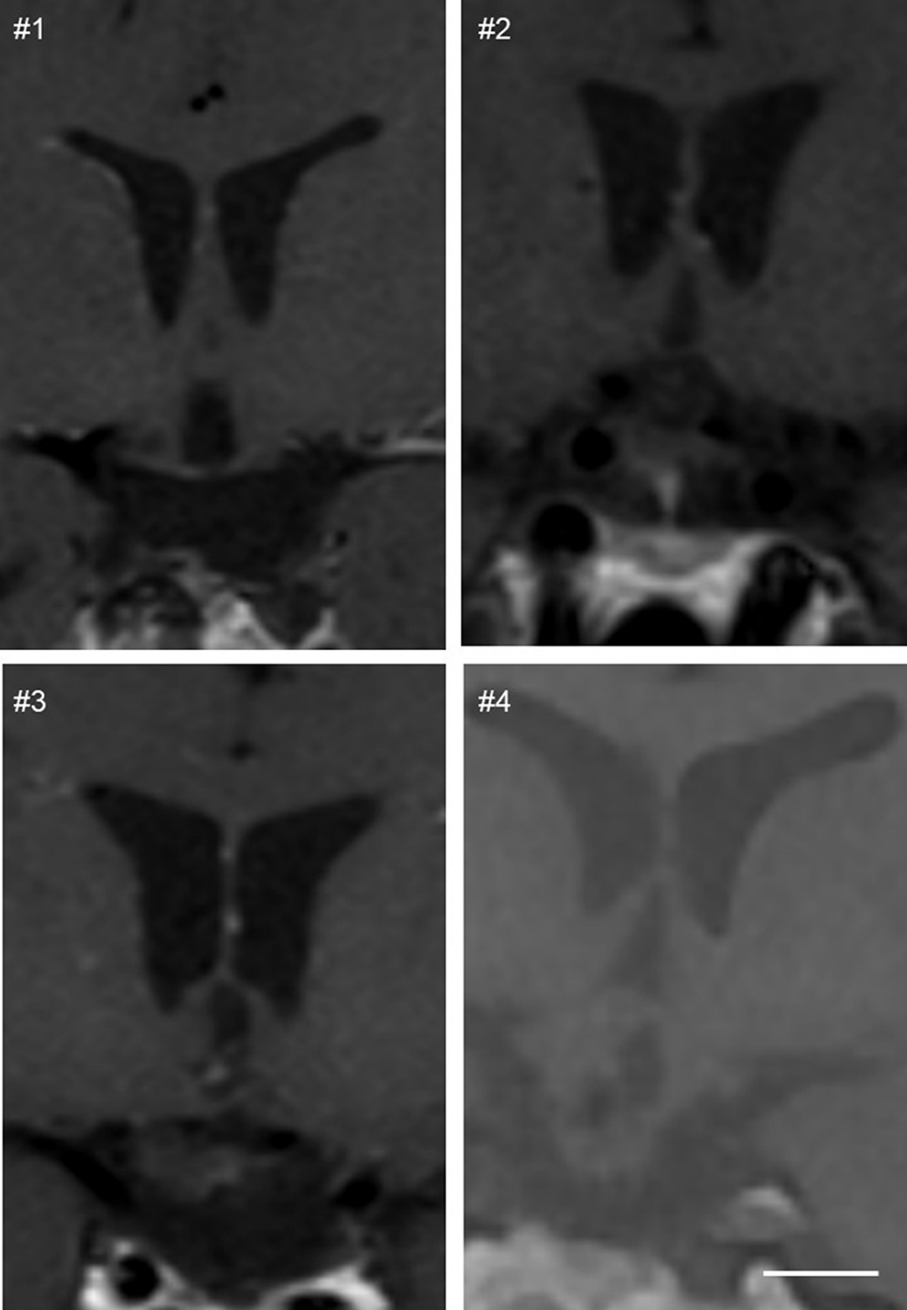
Coronal T1 images from four patients with hypothalamic obesity. The images demonstrate signs of hypothalamic damage, such as dilated asymmetric third ventricle due to hypothalamic atrophy, cystic lesions and damage to the optic chiasm and tuber cinerum

Four female patients with HO were recruited from the outpatient clinic at the department of endocrinology, Aalborg University hospital (Table 1). Inclusion criteria included neuroradiological confirmation of hypothalamic damage (Table 1; Fig. 1) and a continuous increase in body weight post-treatment, despite adhering to a weight loss program.

**Table 1:** Characteristics of patients with hypothalamic obesity following craniopharyngioma resection

	#1	#2	#3	#4
Age (years)	22	69	57	44
Sex	Female	Female	Female	Female
BMI (kg/m^2)^	45.2	55.5	36.0	55.5
Height (cm)	153	163	148	176
Weight (kg)	105.7	147.5	78.8	172.0
Age at diagnosis of CP (years)	6	49	8	7
Histologic sub-type	adamantinomatous	adamantinomatous	adamantinomatous	unavailable
Age at pituitary surgery (years)	6, 10, 12, 14	49	8	7
Age at radiation therapy (years)	14	49	29	7
Hypopituitarism (replacement therapy)	desmopressin hydrocortisone levothyroxine somatropin estradiol	desmopressin levothyroxine somatropin	desmopressin levothyroxine somatropin	desmopressin levothyroxine somatropin

Throughout the outpatient treatment process, there had been a continuous focus on weight loss, including physical activity and diet, albeit with varying degrees of effectiveness. During the initial consultation patients received a dietary counseling session that focused on a balanced calorie-restricted diet. Adherence was routinely assessed at each monthly visit, where semaglutide dose was increased according to general guidelines, body weight recorded and eating behavior assessed via The Three Factor Eating Questionnaire, revised 18-item version (TFEQ). Body composition was assessed by whole body Dual-energy X-ray Absorptiometry-scan (DXA) before initiating treatment and after six months. Biochemical markers including alanine transamine (ALAT), glycated hemoglobin (HbA1c), triglycerides (TG), low-density lipoprotein (LDL), high-density lipoprotein (HDL) and total cholesterol were measured fasting before treatment and after 6 months (Table 4). Written informed consent for publication of their clinical details and/or clinical images was obtained from the patients and data were managed in anonymized form.

**Table 2:** Changes in fat, lean and bone mass (kilograms) measured by DXA scan in four patients with hypothalamic obesity and cranipharyngioma treated for 6 months with semaglutide. DXA = dual-energy X-ray absorptiometry

	Baseline mean (± SD)	After 6 months mean (± SD)	Mean relative change (range)	*P*-value
Total body fat (kg)	58.6 (± 19)	48.5 (± 19)	-17.2% (-12.8; -27.8)	0.006
Truncal body fat (kg)	25.7 (± 7.5)	21.6 (± 9.6)	-16.0% (-9.8; -26.5)	0.02
Upper extremity fat (kg)	7.8 (± 2.6)	6.1 (± 2.2)	-21.8% (-17.6; -25.4)	0.005
Lower extremity fat (kg)	23.4 (± 11)	19.4 (± 10.4)	-17.1% (-12.3; -30.9)	0.006
Total lean mass (kg)	66.9 (± 23)	55.9 (± 17.2)	-16.4% (-11.3; -22.4)	0.05
Truncal lean mass (kg)	32.1 (± 9.6)	27.3 (± 2.3)	-15.0% (-10.3; -19.6)	0.03
Upper extremity lean mass (kg)	6.0 (± 1.7)	5.2 (± 1.4)	-13.3% (1.3; -22.8)	0.13
Lower extremity lean mass (kg)	24.7 (± 12)	19.8 (± 8.0)	-19.8% (-14.3; -26.3)	0.10
Bone mineral density (kg)	1.7 (± 0.3)	1.8 (± 0.4)	2.6% (0.3; 4.6)	0.12

### The three factor eating questionnaire

TFEQ has been validated for use in many populations [16–20] and assesses 3 subtypes of eating behavior; Cognitive restraint (CR), uncontrolled eating (UE) and emotional eating (EE). Generally, higher CR-scores have been associated with a healthier eating behavior, as opposed to high scores of UE and EE [17].

### Statistics

Data were presented as mean ± standard deviation (SD) or range, Student’s paired t-tests were used to compare changes in DXA estimates. A monthly pairwise comparison of the TFEQ questionnaire score (0-100 point scaled scores) was performed using repeated paired t-tests. A p-value < 0.05 was considered statistically significant.

## Results

### Population demographics

All four patients had hypothalamic and pituitary damage due to previous treatment for a craniopharyngioma, including both surgery and radiotherapy (Table 1; Fig. 1). Ages ranged from 22 to 69 years with previous follow-up of 16–49 years, where continuous increases in bodyweight was observed. One patient had class II obesity (36.0 kg/m^2^) and 3 patients had class III obesity (45.2–55.5 kg/m^2^). In all cases, onset of obesity occurred after the diagnosis of hypothalamic lesions and subsequent surgical or radiotherapy treatments. None of our included patients received treatment for, or had previously undiagnosed diabetes mellitus.

**Fig. 2:**
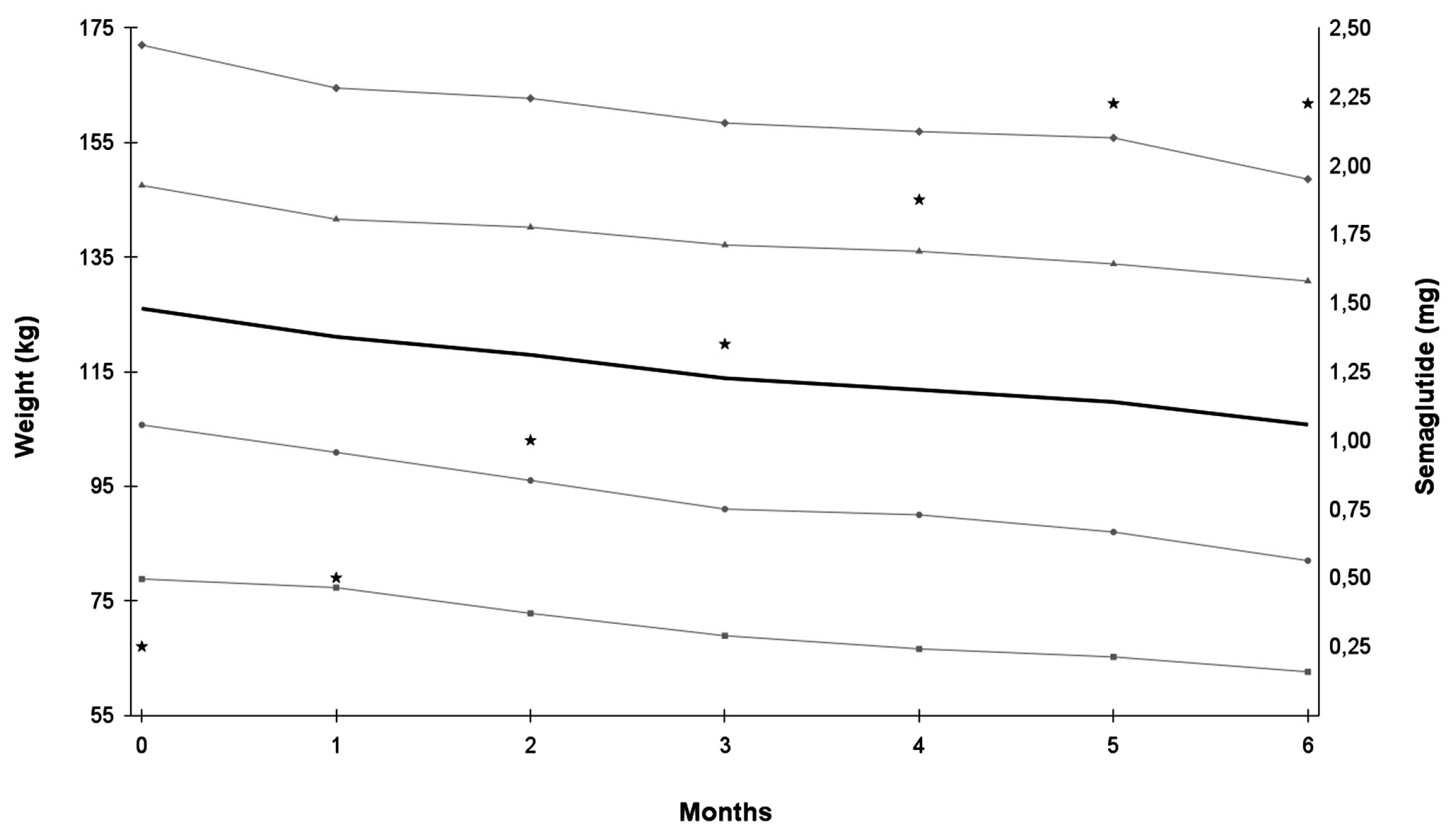
Changes in body weights and semaglutide dosages during a six-month period in four patients with hypothalamic obesity –●– Subject 1, –▲– Subject 2, –■– Subject 3, –♦– Subject 4, — average weight, ★ semaglutide, mean dosage

All participants initiated semaglutide therapy with 0.25 mg once weekly and dosages were increased to their maximally tolerated dose (1.7 or 2.4 mg weekly, Fig. 2). We observed only transient adverse effects including nausea and constipation.

### Body weight

Continuous weight loss was observed in all cases. At baseline, the average weight was 126.0 kg (78.8–172.0 kg), which decreased to 105.8 kg (62.6–148.6 kg) after 6 months of treatment corresponding to a 17.0% (11.3–22.4%) reduction. In terms of BMI (kg/m^2^), this represented a mean reduction of 7.9 (6.7–10.1) from 48.0 (35.0-55.5) at baseline to 40.1 (28.6–48.9) (Fig. 2).

### Body composition

Overall body fat and lean mass decreased by 17.2% (*p* = 0.006, range: -12.8;-27.8%) and 16.0% (*p* = 0.05, range: -11.3;-22.4%), respectively (Table 2). Significant body fat reductions were observed in both the trunk (16.4%, *p* = 0.02, range: -9.8;-26.5%), and the extremities (upper 21.8%, *p* = 0.006, range: -17.6;-25.4%; lower 17.1%, *p* = 0.005, range: -12.3;-30.9%). Lean mass was reduced in the trunk (15.0%, *p* = 0.025, range: -10.3;-19.6%), upper extremities (13.3%, *p* = 0.131, range: +1.3;-22.8%) and lower extremities (19.8%, *p* = 0.096, range: -14.3;-26.3%). Bone mineral density did not change (*p* = 0.12, Table 2).

**Table 3:** Changes in eating behavior at baseline and 1 month (scaled scores) after initiation of semaglutide treatment in four patients with hypothalamic obesity

	Baseline mean (± SD)	After 1 month mean (± SD)	After 1 month mean relative change (range)	*P*-value
Emotional eating	47.2 (± 14.0)	5.6 (± 11.1)	-88.2% (-50.0; -100.0)	*p* = 0.02
Uncontrolled eating	33.3 (± 26.5)	10.2 (± 11.5)	-69.4% (-25.0; -100.0)	*p* = 0.11
Cognitive restraint	50.0 (± 13.4)	55.7 (± 18.7)	11.4% (-50; 114.3)	*p* = 0.73

### Eating behavior

**Fig. 3:**
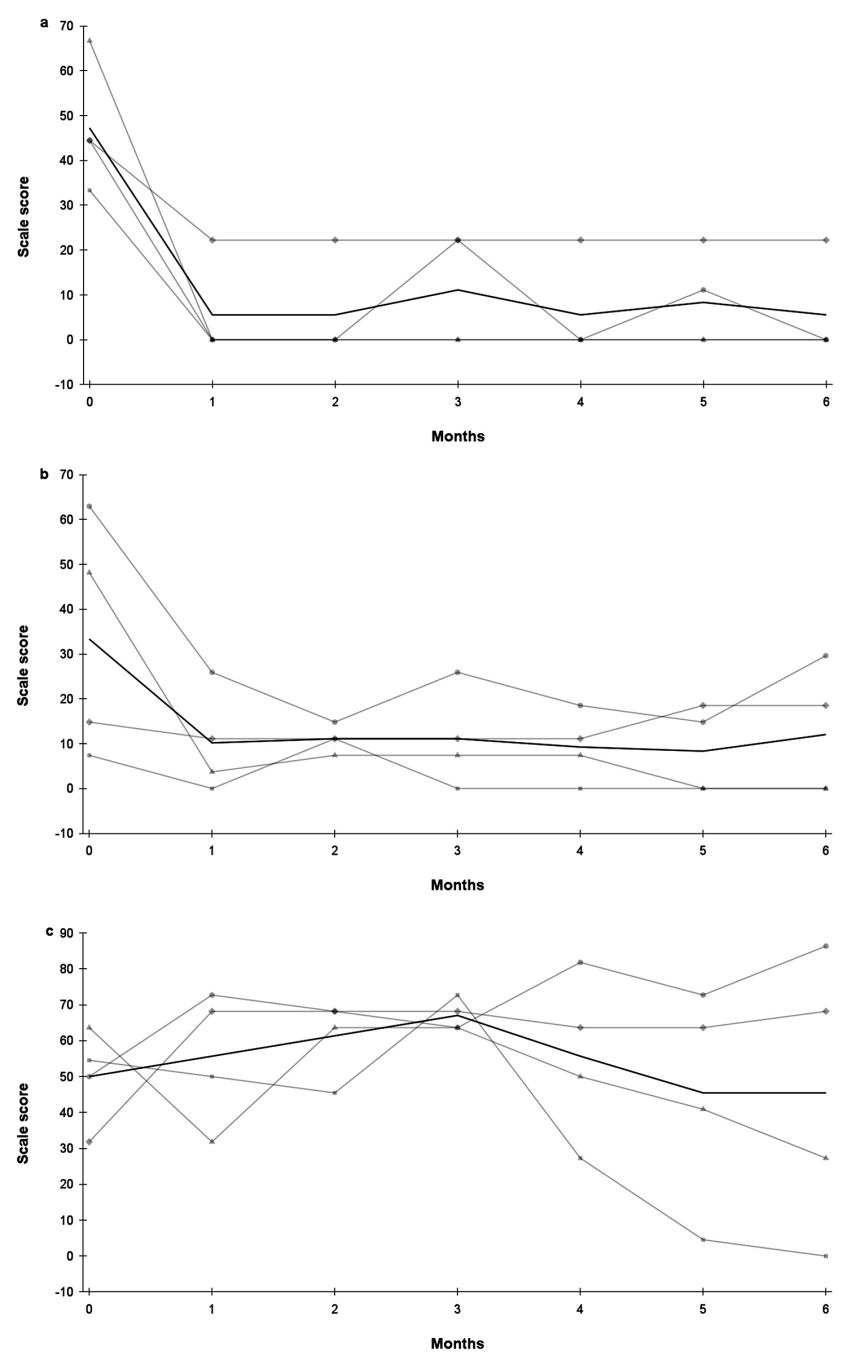
Changes in eating behavior scaled scores during a six-month period of semaglutide treatment in four patients with hypothalamic obesity; 3 A: Emotional eating, 3B: Uncontrolled eating, 3 C: Cognitive restraint, –●– Subject 1, –▲– Subject 2, –■– Subject 3, –♦– Subject 4, — mean

The scaled scores of EE decreased from 47.2 to 5.6% (*p* = 0.02) and for UE from 33.3 to 10.2% (*p* = 0.11) after one month of semaglutide treatment (Table 3; Fig. 3). Both EE and UE scores remained very low during the remainder of follow-up (Fig. 3A and B). The mean CR scores did not significantly change, although intra-personal fluctuation was observed (Fig. 3C).

**Table 4:** Changes in metabolic markers before and after semaglutide treatment. TG = triglycerides, HDL = high-density lipoprotein, LDL = low-density lipoprotein, HbA1c = glaciated hemoglobin, ALAT = alanine transaminase

	Baseline mean (± SD)	After 6 months mean (± SD)	Relative change	*P*-value
TG (mmol/L)	2.0 (± 0.7)	1.2 (± 0.2)	− 41.9% (-14.3; -54.5)	*p* = 0.07
Total cholesterol (mmol/L)	5.1 (± 1.1)	3.6 (± 0.5)	-30.7% (-25.0; -35.1)	*p* = 0.014
HDL (mmol/L)	1.1 (± 0.2)	1.0 (± 0.1)	-13.6% (-8.5; -17.5)	*p* = 0.02
LDL (mmol/L)	2.5 (± 0.7)	2.4 (± 0.8)	-7.1% (-23.5; 32.0)	*p* = 0.64
HbA1c (mmol/mol)	38.5 (± 3.7)	34.0 (± 5.0)	-11.7% (-8.1; -20.6)	*p* = 0.014
ALAT (U/L)	31.0 (± 8.5)	35.5 (± 15.4)	14.5% (-20.0; 64.5)	*p* = 0.47

### Biomarkers

After 6 months of treatment, HbA1c, total cholesterol and HDL had decreased, whereas TG, LDL and ALAT remained unchanged (Table 4).

## Discussion

In our cohort, semaglutide proved to be an effective and safe treatment for HO in craniopharyngioma. We observed positive changes in eating behavior, a considerable weight reduction and improvements in glucose and lipid metabolism.

The effect of exenatide and liraglutide in HO have been examined in several studies, including a recent double-blinded trial of 40 patients, without consistent weight reductions [5, 10, 11, 21, 22]. Semaglutide seems to have a more potent effect on weight loss [13]. All of our patients experienced substantial weight losses in the range of 7 to 10 BMI, in line with the only previous case report to use semaglutide in a patient with HO [13]. We found significant improvements in HbA1c, TG and total cholesterol, which is consistent with findings in persons with non-hypothalamic obesity treated with semaglutide [24]. This has not been clearly demonstrated in studies on earlier GLP1-RA in HO [10, 11], including a systematic review where HbA1c was not or only slightly improved and cholesterol unchanged [21].

Fat loss was evenly distributed, whereas lean mass was only significantly reduced in the trunk and not the extremities. This could be ascribed to the low number of participants, although a relative preservation of lean mass has been reported in obese patients treated with semaglutide, with large reductions in body fat mass [7, 23]. The relative reduction in body mass is in line with our observations of reductions in lean mass of 11–22% and in fat mass of 13–28%. The clinical significance of this, is yet to be studied. Nevertheless, all patients in our cohort reported increased vigor and energy levels, without perceived strength losses. Physical activity was not tracked during the study, but patients reported feeling less limited by their weight and increased activity levels as a consequence of this.

Eating behavior in HO is affected by neuroendocrine dysfunctions due to hypothalamic damage, leading to changes including impaired sensitivity to key hormones, which play critical roles in hunger and satiety signaling [3, 25]. Patients with HO are known to exhibit a higher cognitive restraint as compared to patients with non-hypothalamic obesity, but a comparable uncontrolled eating and hunger score [26]. This is in line with our findings, where major and lasting decreases in EE (88%) were observed after one month of treatment. The same trend was observed for UE, although not statistically significant. These sub-categories refer to eating behaviors that are largely unaffected by physiological hunger cues. The patients reported changes in food choices, including increases in vegetable consumption and reduced cravings of sweets and high-calorie processed foods. All patients reported decreased food focus. Comparable effects of other GLP-1RA on eating behavior in HO have previously been reported and include a reduction in hyperphagia, an increased post-prandial satiety, and an overall decreased food focus [5, 11, 27–31].

## Data Availability

Datasets generated during and/or analyzed during the current study are not publicly available, but are available from the corresponding author on reasonable request.
